# Supplementary material for: Language and family entrepreneurship: empirical research based on micro survey data of CFPS in China
Source: PLoS One. 2025 May 6;20(5):e0318205. doi: 10.1371/journal.pone.0318205 (PMC12054854; doi:10.1371/journal.pone.0318205)
Supplement: Attachment — Submitted filename: Response to Reviewers.docx [file pone.0318205.s003.docx]

**Response to Reviewers**

**About Journal requirements:**

1. Please ensure that your manuscript meets PLOS ONE's style requirements, including those for file naming. The PLOS ONE style templates can be found at

<https://journals.plos.org/plosone/s/file?id=wjVg/PLOSOne_formatting_sample_main_body.pdf> and

<https://journals.plos.org/plosone/s/file?id=ba62/PLOSOne_formatting_sample_title_authors_affiliations.pdf>

**response: Thanks for the editor's reminder. We have made revisions in the manuscript of “the article title” and “the main body” according to the format requirements of PLOS ONE's style guidelines.**

2. When completing the data availability statement of the submission form, you indicated that you will make your data available on acceptance. We strongly recommend all authors decide on a data sharing plan before acceptance, as the process can be lengthy and hold up publication timelines. Please note that, though access restrictions are acceptable now, your entire data will need to be made freely accessible if your manuscript is accepted for publication. This policy applies to all data except where public deposition would breach compliance with the protocol approved by your research ethics board. If you are unable to adhere to our open data policy, please kindly revise your statement to explain your reasoning and we will seek the editor's input on an exemption. Please be assured that, once you have provided your new statement, the assessment of your exemption will not hold up the peer review process.

**response: Thanks for the editor's reminder. We are pleased to provide our research data on this topic.**

3. Please review your reference list to ensure that it is complete and correct. If you have cited papers that have been retracted, please include the rationale for doing so in the manuscript text, or remove these references and replace them with relevant current references. Any changes to the reference list should be mentioned in the rebuttal letter that accompanies your revised manuscript. If you need to cite a retracted article, indicate the article & rsquo;s retracted status in the References list and also include a citation and full reference for the retraction notice.

**response: Thanks for the editor's reminder. We have revised the references according to the format requirements of PLOS ONE's style guidelines.**

**About Reviewers' comments:**

1. Is the manuscript technically sound, and do the data support the conclusions?

The manuscript must describe a technically sound piece of scientific research with data that supports the conclusions. Experiments must have been conducted rigorously, with appropriate controls, replication, and sample sizes. The conclusions must be drawn appropriately based on the data presented.

Reviewer #1: Yes

Reviewer #2: Yes

**response: Thanks.**

2. Has the statistical analysis been performed appropriately and rigorously?

Reviewer #1: Yes

Reviewer #2: Yes

**response: Thanks.**

3. Have the authors made all data underlying the findings in their manuscript fully available?
 The [PLOS Data policy](http://www.plosone.org/static/policies.action#sharing) requires authors to make all data underlying the findings described in their manuscript fully available without restriction, with rare exception (please refer to the Data Availability Statement in the manuscript PDF file). The data should be provided as part of the manuscript or its supporting information, or deposited to a public repository. For example, in addition to summary statistics, the data points behind means, medians and variance measures should be available. If there are restrictions on publicly sharing data & mdash; e.g. participant privacy or use of data from a third party & mdash; those must be specified.

Reviewer #1: Yes

Reviewer #2: Yes

**response: Thanks.**

4. Is the manuscript presented in an intelligible fashion and written in standard English?

PLOS ONE does not copyedit accepted manuscripts, so the language in submitted articles must be clear, correct, and unambiguous. Any typographical or grammatical errors should be corrected at revision, so please note any specific errors here.

Reviewer #1: Yes

Reviewer #2: Yes

**response: Thanks.**

5. Review Comments to the Author

Please use the space provided to explain your answers to the questions above. You may also include additional comments for the author, including concerns about dual publication, research ethics, or publication ethics. (Please upload your review as an attachment if it exceeds 20,000 characters)

Reviewer #1: While the paper excels in many areas, the conclusion could be strengthened. It should include a more comprehensive discussion of the practical implications and contributions of the study. Additionally, it is important to acknowledge the study's limitations, particularly regarding sample size, and to provide suggestions for future research. Addressing these aspects will enhance the overall impact and applicability of the research.

Overall, this manuscript presents a well-conceived and executed study with significant potential to contribute to our understanding of the intersection between language and family entrepreneurship in China. I recommend that the authors address the noted weaknesses in the conclusion to further improve the manuscript.
 Best regards,

**response: Thanks for the reviewer's comments.** **We have made revisions to the conclusion section in three aspects: firstly, we have provided a more detailed explanation of the contribution and practical impact of this study; Secondly, it pointed out the limitations of this study, especially in terms of sample size; Thirdly, it pointed out the further research directions of this study and discussed other aspects of the impact of language on economic performance.**

Reviewer #2: Language and Family Entrepreneurship, a very interesting research topic. The theoretical hypotheses and empirical tests throughout the article are very adequate and scientific.

However, the following comments would hopefully help to improve the quality of the article.

The introduction section needs to clarify the research question(s) of the paper and emphasise the significance of this research topic.
 It is recommended that a discussion section be included before the conclusion section to provide an in-depth account of the theoretical contributions, and practical implications of your research. As you know, the practical implications I saw in your conclusion section, and you can put it in the added discussion section.
 It is necessary to add in the conclusion section the research limitations of the article as well as possible future research directions.
 Good luck!

**response: Thanks for the reviewer's comments. We have addressed the comments as follows:**

**Introduction Section: We have revised the introduction section to strengthen the exposition of the theme and importance of this study, providing a clearer context and rationale for our research questions.**

**Discussion Section: We have added a discussion section before the conclusion to provide a detailed explanation of the research contribution and practical impact of this study, highlighting the significance of our findings.**

**Conclusion Section: We have made revisions to the conclusion section to clarify the limitations of this study, particularly noting the sample size and its potential impact on the generalizability of our findings. Additionally, we have outlined further research directions to explore other aspects of the impact of language on economic performance.**

6. PLOS authors have the option to publish the peer review history of their article ([what does this mean?](https://journals.plos.org/plosone/s/editorial-and-peer-review-process#loc-peer-review-history)). If published, this will include your full peer review and any attached files.
If you choose "no", your identity will remain anonymous but your review may still be made public.
 Do you want your identity to be public for this peer review? For information about this choice, including consent withdrawal, please see our [Privacy Policy](https://www.plos.org/privacy-policy).

Reviewer #1:Yes:Lukman Raimi

Reviewer #2: No

**response: Thanks.**
