# Supplementary material for: Language and family entrepreneurship: empirical research based on micro survey data of CFPS in China
Source: PLoS One. 2025 May 6;20(5):e0318205. doi: 10.1371/journal.pone.0318205 (PMC12054854; doi:10.1371/journal.pone.0318205)
Supplement: Attachment — Submitted filename: Response to Reviewers1213.docx [file pone.0318205.s004.docx]

**Response to Reviewers**

**About Journal requirements:**

Please review your reference list to ensure that it is complete and correct. If you have cited papers that have been retracted, please include the rationale for doing so in the manuscript text, or remove these references and replace them with relevant current references. Any changes to the reference list should be mentioned in the rebuttal letter that accompanies your revised manuscript. If you need to cite a retracted article, indicate the article&rsquo;s retracted status in the References list and also include a citation and full reference for the retraction notice.

**response: Thanks for the editor's reminder. We have thoroughly reviewed and reorganized the references in this article to ensure their completeness, accuracy, and current relevance. All cited references have been verified to confirm that they are not among those that have been retracted.**

**About Reviewers' comments:**

1. If the authors have adequately addressed your comments raised in a previous round of review and you feel that this manuscript is now acceptable for publication, you may indicate that here to bypass the &ldquo;Comments to the Author&rdquo; section, enter your conflict of interest statement in the &ldquo;Confidential to Editor&rdquo; section, and submit your "Accept" recommendation.

Reviewer #2: (No Response)

Reviewer #3: (No Response)

**response: Thanks.**

2. Is the manuscript technically sound, and do the data support the conclusions?

The manuscript must describe a technically sound piece of scientific research with data that supports the conclusions. Experiments must have been conducted rigorously, with appropriate controls, replication, and sample sizes. The conclusions must be drawn appropriately based on the data presented.

Reviewer #2: Yes

Reviewer #3: Yes

**response: Thanks.**

3. Has the statistical analysis been performed appropriately and rigorously?

Reviewer #2: Yes

Reviewer #3: Yes

**response: Thanks.**

4. Have the authors made all data underlying the findings in their manuscript fully available?

The PLOS Data policy requires authors to make all data underlying the findings described in their manuscript fully available without restriction, with rare exception (please refer to the Data Availability Statement in the manuscript PDF file). The data should be provided as part of the manuscript or its supporting information, or deposited to a public repository. For example, in addition to summary statistics, the data points behind means, medians and variance measures should be available. If there are restrictions on publicly sharing data & mdash e.g. participant privacy or use of data from a third party & mdash ; those must be specified.

Reviewer #2: Yes

Reviewer #3: Yes

**response: Thanks.**

5. Is the manuscript presented in an intelligible fashion and written in standard English?

PLOS ONE does not copyedit accepted manuscripts, so the language in submitted articles must be clear, correct, and unambiguous. Any typographical or grammatical errors should be corrected at revision, so please note any specific errors here.

Reviewer #2: Yes

Reviewer #3: Yes

**response: Thanks.**

6. Review Comments to the Author

Please use the space provided to explain your answers to the questions above. You may also include additional comments for the author, including concerns about dual publication, research ethics, or publication ethics. (Please upload your review as an attachment if it exceeds 20,000 characters)

Reviewer #2: It is clearly evident that the authors ought to incorporate a more comprehensive analysis of the practical implications within the discussion section. This enhancement would significantly strengthen the overall impact and applicability of their research findings.

**response: Thanks for the reviewer's comments. In the discussion section of the article, we delve deeper into the implications of our research findings and provide actionable recommendations aimed at enhancing the environment for family entrepreneurship in China. Our recommendations address multiple dimensions, including the cultivation of social relationships, the enhancement of personal capabilities, and the promotion of equitable resource distribution. By addressing these areas, we aim to foster more favorable conditions for entrepreneurship, ultimately leading to improved entrepreneurial outcomes and sustainable business growth.**

**We are grateful for your valuable suggestions, which have been instrumental in refining our research. These modifications enhance the robustness of our analysis and contribute to a deeper understanding of the factors influencing family entrepreneurship in China.**

Reviewer #3: The manuscript under review utilizes CFPS data to explore the relationship between language and entrepreneurial behavior. The topic is innovative, the research design is rational, the methods are appropriately applied, and the conclusions drawn are credible. In the pursuit of academic rigor, the following suggestions are offered for the author's consideration to further refine the paper:

The manuscript focuses on the mechanism by which language influences entrepreneurial behavior and performance. The Sobel-Goodman test is used to examine the mediating roles of cognitive ability and social networks. It is noted that this model is typically used to test the effect of a single mediating variable. It is suggested that the author consider employing a method that allows for the comparison of multiple mediating variables, such as the KHB model. This approach could be beneficial in comparing the relative strengths of different mediators when multiple mediators are present. As the author mentions, human capital and social capital are two significant factors influencing family entrepreneurship. Comparing these two mechanisms within the same framework could potentially enhance the marginal contributions of the study. The paper acknowledges that due to data limitations, speakers of both Mandarin and dialects are not included in the analysis. This highlights the distinction between the concepts of "language ability" and "language use habits." From a typological perspective of language ability, there should be four categories: (weak Mandarin, weak dialect), (weak Mandarin, strong dialect), (strong Mandarin, weak dialect), and (strong Mandarin, strong dialect). However, the paper only addresses two of these categories. If the data does not permit coverage of all such categories, it would be beneficial for the author to discuss the similarities and differences between these concepts in an appropriate section of the paper.

It is hoped that these suggestions will be helpful in improving the manuscript.

Best regards，

**response: Thanks for the reviewer's comments. Modifications Based on Your Feedback.**

**firstly, In response to your feedback, we have adopted the Karlson, Holm, and Breen (KHB) mediation analysis method to systematically decompose and statistically evaluate the pathways through which family daily language influences family entrepreneurial behavior. Building upon the basic model, we introduced two mediating variables: social network (social) and cognitive ability (cog). This allowed us to compare and analyze the impact and contribution strength of these mechanisms within a unified analytical framework.**

**Secondly, We have carefully distinguished between "language ability" and "language habits." After thorough consideration, we propose categorizing language ability into three distinct groups:(Weak Mandarin, Strong Dialect), (Strong Mandarin, Weak Dialect), (Strong Mandarin, Strong Dialect). For language habits, we suggest a binary classification: (Weak Mandarin, Strong Dialect) and (Strong Mandarin, Weak Dialect). The distinction between language proficiency and language habits is crucial for understanding the complex interplay between linguistic factors and entrepreneurial outcomes. While language ability reflects an individual's competence in using a language, language habits reveal how this ability is actually put into practice in daily life. By considering these two dimensions, we can gain a more comprehensive understanding of how language influences family entrepreneurship in China.**

**Finally,** **Due to data limitations, this study adopts language usage habits as the primary research classification. This approach allows us to explore the practical implications of language choices in everyday life, which are particularly relevant to family business dynamics and social interactions. If more detailed data become available in the future, we hope to conduct further research that incorporates both language ability and language habits to provide a more nuanced analysis.**

**We are grateful for your valuable suggestions, which have been instrumental in refining our research. These modifications enhance the robustness of our analysis and contribute to a deeper understanding of the factors influencing family entrepreneurship in China.**

7. PLOS authors have the option to publish the peer review history of their article (

what does this mean?). If published, this will include your full peer review and any attached files. If you choose &ldquo;no&rdquo;, your identity will remain anonymous but your reviewmay still be made public.

Do you want your identity to be public for this peer review?

For information about this choice, including consent withdrawal, please see our Privacy Policy。

Reviewer #2: No.

Reviewer #3:

Yes: Xinyan Xie[NOTE: If reviewer comments were submitted as an attachment file, they will be attached to this email and accessible via the submission site. Please log into your account, locate the manuscript record, and check for the action link "View Attachments". If this link does not appear, there are no attachment files.]

**response: Thanks.**
